# Differential reactivity of closely related zinc(II)-binding metallothioneins from the plant *Arabidopsis thaliana*

**DOI:** 10.1007/s00775-017-1516-6

**Published:** 2017-12-07

**Authors:** Hasan T. Imam, Claudia A. Blindauer

**Affiliations:** 1 Department of Chemistry, The University of Warwick, Coventry, CV4 7AL UK; 2 School of Chemistry, University of St. Andrews, St. Andrews, KY16 9ST UK

**Keywords:** Nuclear magnetic resonance, Mass spectrometry, Zinc, Metallothionein, Metal homeostasis

## Abstract

The dynamics of metal binding to and transfer from metalloproteins involved in metal homeostasis are important for understanding cellular distribution of metal ions. The dicotyledonous plant *Arabidopsis thaliana* has two type 4 seed-specific metallothionein homologues, MT4a and MT4b, with likely roles in zinc(II) homeostasis. These two metallothioneins are 84% identical, with full conservation of all metal-binding cysteine and histidine residues. Yet, differences in their spatial and temporal expression patterns suggested divergence in their biological roles. To investigate whether biological functions are reflected in molecular properties, we compare aspects of zinc(II)-binding dynamics of full-length MT4a and MT4b, namely the pH dependence of zinc(II) binding and protein folding, and zinc(II) transfer to the chelator EDTA. UV–Vis and NMR spectroscopies as well as native electrospray ionisation mass spectrometry consistently showed that transfer from Zn_6_MT4a is considerably faster than from Zn_6_MT4b, with pseudo-first-order rate constants for the fastest observed step of *k*_obs_ = 2.8 × 10^−4^ s^−1^ (MT4b) and *k*_obs_ = 7.5 × 10^−4^ s^−1^ (MT4a) (5 µM protein, 500 µM EDTA, 25 mM Tris buffer, pH 7.33, 298 K). 2D heteronuclear NMR experiments allowed locating the most labile zinc(II) ions in domain II for both proteins. 3D homology models suggest that reactivity of this domain is governed by the local environment around the mononuclear Cys_2_His_2_ site that is unique to type 4 MTs. Non-conservative amino acid substitutions in this region affect local electrostatics as well as whole-domain dynamics, with both effects rendering zinc(II) ions bound to MT4a more reactive in metal transfer reactions. Therefore, domain II of MT4a is well suited to rapidly release its bound zinc(II) ions, in broad agreement with a previously suggested role of MT4a in zinc(II) transport and delivery to other proteins.

**Electronic supplementary material:**

The online version of this article (10.1007/s00775-017-1516-6) contains supplementary material, which is available to authorized users.

## Introduction

The unique importance of the essential micronutrient zinc(II) to the health of all organisms is increasingly recognised [1], with the number of confirmed and predicted zinc(II)-requiring proteins still on the rise [2]. The pervasive utilisation of zinc(II) in biological systems renders it a so-called type 2 nutrient [3], which means that its deficiency impacts on a multitude of physiological processes. This is not only true for animals and man [4–6], but also for plants [7, 8].

Although raising awareness of the prevalence and far-reaching consequences of (human) zinc deficiency has been a long and slow process [9], zinc deficiency is now recognised as one of the most frequent micronutrient deficiencies [10]. It is estimated that between a sixth and a third of the world’s population is at risk of inadequate zinc(II) intake [11, 12], especially where diets are largely plant-based [10]. This staggering impact has been recognised by policymakers in the 2008 and 2012 Copenhagen Consensus conferences [13], and has meanwhile fostered practical initiatives and programmes such as HarvestPlus [14]. The latter is aimed at increasing the nutritional value of edible crops through the traditional breeding approaches—in other words, biofortification [15–18]. However, zinc(II) accumulation by plants is not only critical to combat human zinc deficiency, but also for food security in general, because insufficient zinc(II) uptake by plants, which is estimated to occur on 162 MHa of arable land, severely limits crop yields [10]. For these reasons, research into the molecular details of zinc(II) trafficking in plants has intensified throughout the past decade, leading to the identification of scores of membrane-bound zinc(II) transporters [19–22]. Particular focus has been given more recently to zinc(II) trafficking into seeds [23–25], with support from metallomics-type imaging [26] and speciation studies [27].

It has been argued recently that enzyme-bound zinc(II) is now reasonably well understood, but that the nature of “mobile” zinc(II) in biological systems has remained largely mysterious [28]. In particular, and despite major progress in elucidating assembly pathways for many metalloproteins that depend on other essential metals, it is still unclear how the thousands of nascent zinc(II)-requiring proteins actually acquire their metal co-factor. It is now, however, well established that the extremely low concentrations of “free” zinc(II) in cytosols [29] are insufficient to permit this process to occur at reasonable rates, and that ligands with appropriate affinities and fast zinc(II) binding kinetics must play a role. Metallothioneins (MTs)—small, cysteine-rich proteins that bind several metal ions in typical metal–thiolate clusters—have long been proposed as such near-ubiquitous bioligands that fulfil these criteria [30, 31]. A number of in vitro and in vivo studies have demonstrated that MT-bound zinc(II) can be transferred to zinc(II)-requiring proteins [32–34].

Flowering plants express up to four different types of MTs [35–37], with the types defined by the number and spacing of Cys residues. After the discovery of MTs in plants in the late 80s [38, 39], relatively little work on the protein level was accomplished, but the past decade has seen a welcome and steady proliferation of biophysical studies on plant MT proteins. Spectroscopic data and information on metal stoichiometry are available for types 1–3 [40–44], but the best-studied plant MT is the type 4 MT E_C_-I/II from wheat [45–49].

**Fig. 1:**
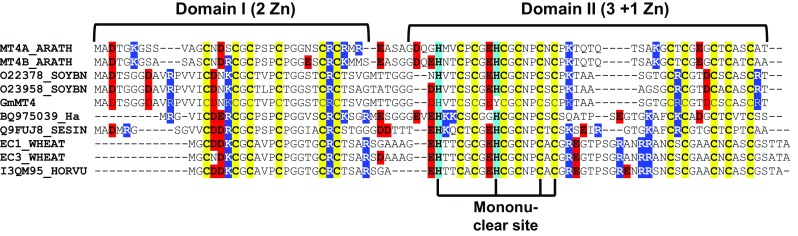
Sequences of *Arabidopsis thaliana* MT4a and MT4b (rows 1 and 2), aligned with other representative type 4 MT mentioned in the text. The sequence labelled GmMT4 refers to soybean MT4, and BQ975039_Ha to sunflower MT4, both reported in [53]. All other sequences are retrieved from UniProt and refer to MT4 homologues from soybean (O22378_SOYBN and O23958_SOYBN), sesame (Q9FUJ8_SESIN), wheat (EC1_WHEAT and EC3_WHEAT), and barley (I3QM95_HORVU). Cys residues are highlighted in yellow; His residues in cyan. Charged residues are highlighted in red (Asp and Glu), and blue (Lys and Arg). Domain organisation and the residues forming the mononuclear Cys_2_His_2_ site are also indicated

Type 4 MTs (Fig. 1) are also of particular interest in the context of seed zinc(II) homeostasis: they are almost exclusively expressed in developing seeds, where their expression is highly upregulated during embryogenesis and seed maturation [37, 50, 51], and the proteins are typically isolated with varying amounts of zinc(II) bound from either native or recombinant hosts [45, 46, 52, 53]. Wheat E_C_ was isolated from wheat germs in its fully metallated form, with a dominating Zn_6_ species, but a M_7_ species that included copper was also observed [39, 45]. It is unknown whether the copper was incorporated into the protein in planta or during purification. Recombinant expression in *E. coli* typically yields metal-saturated MTs, although exceptions are known [37].

Furthermore, their heterologous expression in yeast [54] or *E. coli* [55] and their ectopic expression in plants [55] lead to cellular zinc(II) accumulation. Silencing of MT4 genes in *A. thaliana* led to lower zinc(II) contents in seeds, which was accompanied by growth inhibition that could be rescued by elevated zinc(II) supply. In turn, overexpression of these genes led to larger seeds and more vigorous seedling growth [55]. In contrast to most other MTs, the expression of type 4 plant MTs is not metalloregulated, but is governed by plant hormones [37]. Type 4 MTs are unlikely to play a role in metal toxicity, although some studies have found that they are able to confer zinc(II) or copper tolerance when expressed heterologously [54] or ectopically [56]. The regulation by abscisic acid points towards a role in desiccation and/or rehydration, and it is likely that especially the latter process may require the supply of zinc(II) to newly synthesised proteins. Evidently, a prerequisite of zinc(II) donation during rehydration requires zinc(II) storage before or during desiccation, and it is an attractive proposition that type 4 MTs are important in both of these processes.

The primary sequences of type 4 MTs comprise three Cys-rich stretches with 6, 6, and 5 Cys residues, respectively. These three stretches are separated by relatively short Cys-free regions. In addition, two histidine residues in the second Cys-rich stretch are almost fully conserved in flowering plants [45]. Wheat E_C_-I/II is the only plant MT for which 3D structures are available [57–59]. All 17 cysteines and the two histidines bind six zinc(II) ions in two separate domains, with all zinc(II) ions having tetrahedral coordination spheres. The six cysteines of the N-terminal γ-domain (domain I in Fig. 1) form a Zn_2_Cys_6_ binuclear cluster with two bridging thiolates. The remaining 11 cysteines and the 2 histidines in the second domain (domain II, also termed the beta(E) domain [57]) bind a total of four zinc(II) ions. Type 4 MTs are unique amongst MTs in that they contain a mononuclear Cys_2_His_2_ site; in wheat E_C_, this is formed by His32, His40, Cys46, and Cys48.^1^ The remaining three zinc(II) ions are bound to nine cysteines; in the published 3D structure, these have been modelled as a six-membered ring consisting of the three zinc(II) ions and three bridging thiolates, with each zinc(II) ion also bound to two terminal thiolates—in analogy to the M_3_Cys_9_ clusters found in mammalian and several other MTs from animals [46, 60]. Correct population of the mononuclear site with a zinc(II) ion is absolutely critical to ensure an ordered structure and defined metal-loading for the entire domain II [61].

*Arabidopsis thaliana*, a dicotyledonous plant, has two type 4 MT homologues^2^ amongst its seven active MT genes [62]. MT4a and MT4b contain 83 and 84 amino acid residues, respectively, in their primary protein sequences (Fig. 1). Like their E_C_ homolog from wheat, they comprise 17 cysteines and two histidines and bind six zinc(II) ions [37]. It appears that these proteins are particularly important in the early stages of germination and seedling growth, when the young plant has to rely on its internal zinc(II) stores to populate zinc(II)-requiring proteins [55]. Indeed, the mRNA levels for several zinc(II)-requiring transcription factors and enzymes were reduced in *A. thaliana* plants with co-silenced MT4 genes [55]. Intriguingly, although both homologues are exclusively expressed in developing seeds, reporter assays have revealed that they differ in their temporal and spatial expression patterns: MT4a expression in the plant embryo starts later and remains restricted to vascular tissues, whilst MT4b expression starts earlier, is overall higher and evenly distributed throughout all embryonic cells [55]. This has led to the suggestion that the biological functions of these two closely similar MT4 homologues (84% sequence identity on the amino acid level) are likely to differ, with MT4a being involved in metal transport and MT4b in metal storage—although we note that ultimately, all stored zinc(II) will have to be mobilised.

The general concept that biophysical properties of a protein should be correlated with their biological function is also relevant to MTs [61–66]. This is particularly apparent for MTs from organisms where more than one homologue is active, as shown for *C. elegans* MTL1 and MTL2 [67, 68], mammalian MT1 and MT2 [69], sea urchin SpMTA and SpMTB [70], and snail MTs [71]. Most of the latter studies have focused on metal specificity; the *A. thaliana* MT4 system offers the opportunity to compare the binding properties for a single metal—zinc(II)—of two closely similar proteins from the same biological system. Combining electrospray ionisation mass spectrometry (ESI-MS) and nuclear magnetic resonance (NMR) spectroscopy, we have studied proton-induced zinc(II) loss to correlate speciation with protein folding. Second, in the light of the suggested different biological functions, and the idea that MTs may directly transfer zinc(II) to other proteins [32–34], we were particularly keen to explore the propensity of MT4a and MT4b to transfer zinc(II), and have used the small-molecule metal chelator EDTA to probe this.

## Experimental

### Protein expression and purification

Expression and purification of these two homologues in their unlabelled forms were previously described by our group [36]. In brief, pET-based plasmids containing the coding regions for MT4a and MT4b, kindly provided by Peter Goldsbrough, were used to transform *E. coli* cells (Rosetta 2(DE3)pLysS; Novagen). Protein expression was induced by 0.5 mM isopropyl-β-d-1-thiogalactoside (IPTG), and at the same time, the medium was supplemented with 0.5 mM ZnSO_4_. The cell lysate was fractionated by chemical precipitation using a 100:8 (v/v) ethanol:chloroform mixture [72]. The precipitated protein(s) were redissolved in 20 mM ammonium bicarbonate buffer (pH 7.8) and purified by gel filtration chromatography (GE Healthcare Superdex G75 16/60 HiLoad column mounted on an ÄKTA Purifier 10). Labelled proteins for NMR spectroscopy were expressed in M9 media containing either ^15^NH_4_Cl or ^13^C-glucose/^15^NH_4_Cl as sources of ^15^N for singly or ^13^C/^15^N for doubly labelled samples using standard protocols. Labelled protein expression was induced at OD_600_ = 1.0–1.1, using 0.5 mM IPTG, with supplementation of 0.5 mM ZnSO_4_. The cells were allowed to grow for 14 h at 20 °C at 180 rpm, before harvesting by centrifugation at 5000×*g* for 15 min at 4 °C.

### Determination of protein concentration and metal–protein stoichiometry

Inductively Coupled Plasma-Optical Emission Spectroscopy (ICP-OES) (Perkin-Elmer Optima 5300 DV, Model S10) was used to determine the S, Zn, Cd, and Cu contents of the purified proteins as described previously [73, 74]. In addition, the protein concentration was also routinely determined from the concentration of free thiols (cysteine) in the EDTA-demetallated proteins by Ellman’s reagent, 5,5′-dithio-bis(2-nitrobenzoic acid) (DTNB) at 412 nm by UV–visible spectroscopy using *N*-acetyl-cysteine as standard (Biomate 3 spectrophotometer, Fisher Scientific, UK). Typically, ICP-OES and UV–Vis results agreed within 10%.

### UV–visible spectroscopy: Zn transfer to EDTA

The metal chelator EDTA [Ethylenediaminetetraacetic acid; 2,2′,2″,2‴-(ethane-1,2-diyldinitrilo)tetraacetic acid; Sigma-Aldrich, UK] was used to study the metal transfer dynamics of MT4a and MT4b by UV–Vis spectroscopy. Zn_6_MT4a or Zn_6_MT4b (5 µM, 0.7 mL, 25 mM Tris buffer, pH 7.33) was reacted with EDTA at different concentrations (0.05, 0.25, 0.5 mM, 25 mM Tris buffer, pH 7.33) at 298 K. Metal release was monitored as a change in absorption at 220 nm. The blank solution contained an equivalent concentration of EDTA in the same reaction buffer. Changes in absorbance were monitored for 3 h. All UV–visible experiments were carried out using a Cary 50 (Varian) spectrophotometer. Data were processed in Microsoft Excel and plotted using Origin Pro 9.1.

### Mass spectrometry for observation of metallospecies at different pH values and during reaction with EDTA

The proteins were desalted and buffer-exchanged (10 mM NH_4_HCO_3_, pH 7.8) using PD-10 columns (Sephadex G25, GE Healthcare). The protein eluates were concentrated using Amicon Ultra-4 (3000 MWCO, Millipore) filter devices.

To observe speciation at different pH values, desalted aliquots of Zn_6_MT4a and Zn_6_MT4b (25 µM, 200 µL, 10 mM NH_4_HCO_3_, pH 7.8) were acidified with small volumes of different concentrations of formic acid (0.1–5 M). Mass spectra of the samples (25 µM, 10% v/v CH_3_OH, 10 mM NH_4_HCO_3_ buffer, pH 7.8–2.4) were recorded. To avoid salt contamination from the KCl solution of the glass electrode (Hamilton Biotrode pH electrode), each sample was divided into two aliquots and the pH was measured before (aliquot 1, discarded) and after (remainder of aliquot 2) acquisition of mass spectra. The pH values given are not corrected for the 10% CH_3_OH content.

For observation of speciation during reaction with EDTA, desalted solutions of Zn_6_MT4a or Zn_6_MT4b (270 µM, 10 mM NH_4_HCO_3_ buffer, pH 7.8) were reacted with six molar equivalents of EDTA (1620 µM in 10 mM NH_4_HCO_3_; pH adjusted to 7.8 with NH_3_, 298 K) for up to 22 h. At different time intervals, aliquots were taken and diluted (25 µM, 10% v/v CH_3_OH, 10 mM NH_4_HCO_3_ pH 7.8) prior to recording mass spectra. The pH value after the reaction was checked and found to have not changed significantly.

All mass spectra were recorded on a Bruker Daltonics MicroTOF mass spectrometer fitted with an electrospray ionisation source operating in positive mode. The samples were injected into the spectrometer by a syringe pump with a flow rate of 240 μL h^−1^. Other parameters were fixed as: temperature 195 °C, nebulizer 0.6 bar, dry gas 4.5 L min^−1^, capillary exit 100 V, skimmer1 50 V, skimmer2 25.2 V, hexapole1 24.2 V, hexapole2 22.4 V, hexapole RF 450 V, transfer time 81 μs, and detector TOF 2300 V. The data were recorded over 0.4–2 min for a range of 500–5000 *m*/*z*. The experimental data were then smoothed and deconvoluted using Bruker Compass DataAnalysis v. 4 (Bruker Daltonik, Germany).

### Nuclear magnetic resonance (NMR) spectroscopy

#### Sample conditions, NMR data acquisition, and processing

All protein samples (various concentrations) were prepared in a standard buffer, in the following referred to as “NMR buffer” (50 mM Tris-D_11_, 10% D_2_O, 50 mM NaCl) at different pH values (vide infra). Homonuclear ^1^H spectra were acquired on unlabelled samples for Zn_6_MT4a and Zn_6_MT4b. Heteronuclear 2D and 3D [^1^H, ^15^N] spectra were obtained for ^15^N-labelled samples for Zn_6_MT4a and Zn_6_MT4b, and a ^13^C- and ^15^N-labelled sample of Zn_6_MT4b was used to record triple-resonance data (HNCA and HN(CO)CA) to facilitate sequential assignment.

Experiments were carried out using a Bruker Avance 700 Ultrashield spectrometer mounted with a TCI cryoprobe. Spectra were recorded at an operating frequency of 700.24 MHz for ^1^H, 70.95 MHz for ^15^N, and 176.08 MHz for ^13^C. The residual water resonance was used as a reference for ^1^H chemical shifts; ^13^C and ^15^N are referenced externally to DSS (2,2′-dimethyl-2-silapentane-5-sulfonate) and ^15^NH_4_Cl, respectively. All NMR spectra were acquired at 298 K. Recorded spectra were processed using Bruker Topspin v. 2.1 software. For 2D and 3D spectra, phase- and baseline-corrected Topspin files were transferred to Sparky v. 3.106 [75] for analysis.

1D ^1^H-NMR spectra were recorded with 16k complex data points, 128 scans and a spectral width of 15.9 ppm. 2D [^1^H, ^1^H] Total Correlation (TOCSY, mixing time of 60–65 ms) and Nuclear Overhauser Enhancement (NOESY, mixing time of 60 or 100 ms) spectra were recorded with 16 scans, 4k data points in F2 and 512 or 400 increments in F1 with a spectral width of 16 ppm centred at the water peak at ~ 4.7 ppm. A squared sine-bell function was used to apodize the raw data prior to Fourier transformation with 2k × 2k data points. 2D heteronuclear single quantum coherence [^1^H, ^15^N] HSQC spectra for Zn_6_MT4a and Zn_6_MT4b used for assignment purposes were recorded with 4k data points in F2 (^1^H) and 128 increments in F1 (^15^N). Spectra were acquired with a spectral width of 16 ppm in F2 and 40 ppm in F1 over 16 scans with a ^1^ *J*_NH_ coupling constant of 90 Hz. The raw data were apodized using a squared sine-bell function and Fourier transformed into 4k × 512 data points in the F2 × F1 dimensions, respectively.

3D [^1^H, ^1^H, ^15^N] TOCSY-HSQC (mixing time of 60 ms) and NOESY-HSQC (mixing time of 100 ms) spectra were acquired with spectral widths of 14 ppm for F3 (^1^H), 32 ppm for F2 (^15^N) and 14 ppm for F1 (^1^H) with 16 scans. Spectra were acquired with 2048 data points in F3 (^1^H), 36 increments for F2 (^15^N), and 128 increments for F1 (^1^H). HNCA and HN(CO)CA spectra were obtained with 2048 data points in F3 (^1^H), 40 increments for F2 (^15^N), and 64 increments for F1 (^13^C) with 16 scans. Spectral widths were 14.0 ppm for F3 (^1^H), and 32 ppm for both F2 (^15^N) and F1 (^13^C). The data were apodized and Fourier transformed into 2k(F3) × 64(F2) × 512(F1) data points for [^1^H, ^1^H, ^15^N] TOCSY-HSQC and 2k(F3) × 64(F2) × 256(F1) data points for [^1^H, ^1^H, ^15^N] NOESY-HSQC, HNCA and HN(CO)CA.

#### 1D ^1^H NMR spectroscopy: pH dependence and reactions with EDTA

For experiments to study the pH dependence of protein folding, the pH of Zn_6_MT4a or Zn_6_MT4b samples (400 µM, NMR buffer, pH 8.48) was adjusted with incremental additions of acid (0.1–1 M HCl). Samples were allowed to equilibrate for ca. 10 min prior to recording 1D ^1^H-NMR spectra. Trimethylsilyl propionate (TSP) was used as an internal standard. In each case, the pH was measured before and after NMR data acquisition. To allow rapid monitoring of protein folding during reaction with EDTA, Zn_6_MT4a or Zn_6_MT4b (277 µM, NMR buffer, pH 7.4) was mixed with an equimolar amount of EDTA [1:6 with respect to (protein), NMR buffer, pH 7.4]. Immediately after mixing a series of 1D, ^1^H-NMR spectra were recorded. The pH value after the reaction was checked and found to have not changed significantly.

#### [^1^H, ^15^N] HSQC NMR spectroscopy: reaction with EDTA

Homogeneously labelled (^15^N) samples of Zn_6_MT4a or Zn_6_MT4b (251 and 267 µM, NMR buffer, pH 7.43) were mixed with EDTA (1:6 with respect to [protein], NMR buffer, pH 7.4, 298 K). Immediately after mixing, a series of 2D [^1^H, ^15^N] HSQC spectra (4k × 64 datapoints over 4 scans; 320 s total duration per experiment) were recorded. The pH value after the reaction was checked and found to have not changed significantly.

### Homology models and electrostatic potentials

Structural models were generated using Modeller v. 9.13 [76]. The solution structures of the separate domains of zinc(II)-loaded wheat E_C_ (PDB 2L62 [58] and 2KAK [57]) were used as templates. Side-chain conformations of Modeller-generated models were optimised using the program Scwrl v. 4.0 [77]; however, side-chain conformations of metal coordinated residues (Cys and His) were kept as obtained from the Modeller runs. Finally, Zn ions were manually incorporated into the Scwrl4 optimised model, and structures were energy-minimised using the Amber ffSB14 force field incorporated in the program Chimera v. 1.11. Generated models were validated using the WHATIF server [78]. Electrostatic surface potentials were calculated using the APBS (Adaptive Poisson–Boltzmann solver) plugin of PyMol v. 1.8, with the underlying partial charges calculated using PDB2PQR v. 2.0.0 [79].

## Results and discussion

We have previously expressed and purified the MT4a and MT4b proteins from *A. thaliana* [37]. The determination of the masses of the apo proteins by ESI-MS confirmed cleavage of the N-terminal initiator methionine residue. ESI-MS at neutral pH and inductively coupled plasma-optical emission spectroscopy (ICP-OES) also confirmed that our expression and purification protocols yield overwhelmingly the Zn_6_ species in both cases; this was confirmed for each individual batch used in the studies described below.

### Sequential assignment of *A. thaliana* MT4a and MT4b

**Fig. 2:**
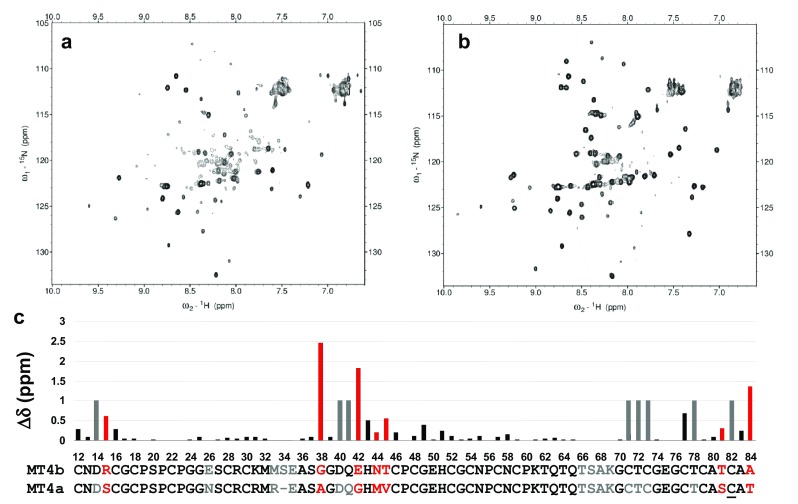
Protein folding, chemical shift assignment, and comparison of *A. thaliana* MT4a and MT4b. **a** 2D [^15^N, ^1^H] HSQC spectrum of Zn_6_MT4a, **b** 2D [^15^N, ^1^H] HSQC spectrum of Zn_6_MT4b **c** Weighted differences (∆δ = (∆δ(^1^H)^2^ + (1/7 × ∆δ(^15^N))^2^)^1/2^ between backbone N–H chemical shifts between MT4a (residues 12–83) and MT4b (residues 12–84). The sequences of MT4a and MT4b, as well as the corresponding bars in the chart are colour-coded to highlight non-assigned residues (grey; in cases where assignments were only possible in one homologue, an arbitrary value of ∆δ = 1 was allocated to highlight this difference) and residues that differ between the two proteins (red) (also see “Discussion”). Cys81 (underlined) in MT4a was only observed in 2D ^1^H spectra

To enable comparisons between the two MT homologues on a structural level, sequential NMR assignments were obtained for both Zn_6_MT4a and Zn_6_MT4b (see Supplementary Table S1 for chemical shift lists). Most residues lying within the two domains were assigned for both proteins (Fig. 2); this indicates that both domains of both proteins were reasonably well folded—with some exceptions that will be discussed later on. The 11 N-terminal residues (Fig. 1) could not be assigned for either protein. Extended N-termini are a feature of some type 4 MTs from dicotyledonous plants [36]. Our inability to assign these residues for either MT4a or MT4b suggests that these stretches are largely unstructured and flexible. Indeed, given that the structure of wheat E_C_ domain I (also termed the γ domain) is known [58], it is evident that the N-terminal extension of certain dicot MT4s is unlikely to be required for protein folding. Other residues that are missing in our assignments are two (MT4a) or three (MT4b) residues in the region that links the two domains, and four residues in the Cys-free loop within domain II, in each case indicating high structural flexibility. Several further residues in domain II of MT4a were also not assignable, including Cys70 and Cys81 (see Supplementary Table S1 for details). The NMR assignments will be used in the following to rationalise the effects of proton- and chelator-induced metal loss on protein folding.

### Proton-induced metal loss: ESI-MS and NMR studies

An important property that is often used to distinguish MTs from other metal-binding proteins is the comparatively low pH at which their bound metal ions succumb to displacement by protons [40, 80]. We have previously determined the pH of half displacement [pH(1/2)] of *A. thaliana* MT4a (4.55) and MT4b (4.56) by UV–Vis spectroscopy, finding that by this measure, their overall pH stability was essentially the same [36], and also did not differ significantly from that of wheat E_C_ determined under the same conditions (4.53 [81]).

**Fig. 3:**
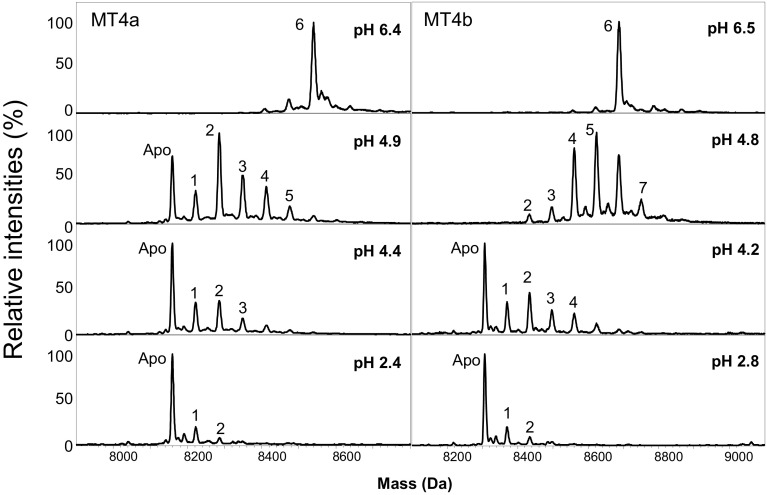
Representative deconvoluted mass spectra at different pH values (25 µM protein, 10 mM NH_4_HCO_3_, 10% MeOH). The neutral masses of 8539.2 (theoretical mass 8539.07) and 8685.5 (theoretical mass 8686.07) correspond to full-length MT4a or MT4b (without N-terminal Met), respectively, with 6 Zn^2+^ ions bound

The determination of pH(1/2) values is a quick but relatively coarse measure to characterise MTs; more insight may be gained by ESI-MS, which is the only method capable of providing information on which species are present under the given conditions [82–87]. ESI-MS spectra of Zn_6_MT4a and Zn_6_MT4b at different pH values (Fig. 3) confirm that at a pH well above the pH(1/2), both homologues show a clearly dominating Zn_6_, i.e., the fully metallated species.

Intriguing differences were captured at pH values close to the pH(1/2): whilst the spectrum for MT4b at pH 4.8 was dominated by Zn_5_, Zn_4_, and Zn_6_ species, the most abundant species for MT4a at pH 4.9 was Zn_2_ followed by the apo form. No apo form was observed at pH 4.8 for MT4b, but surprisingly, a new over-metalled Zn_7_ species emerged. Over-metallated species have been observed previously for a range of MTs, including human MT1A [60, 88, 89] and MT3 [90, 91], and wheat E_C_ [57]. In each of these cases, the over-metallated species were observed after addition of excess metal. Metal redistribution upon a change in pH similar to that observed here was also evident for Cd-loaded earthworm MT2 [64], where the over-metalled Cd_8_ species emerged at pH 3.5, alongside several under-metallated species. The significance of these species is unclear.

Further lowering the pH to 4.4 for MT4a resulted in formation of apo-MT4a as dominating species, along with under-metallated species of Zn_1_, Zn_2_, Zn_3_, and a very low abundance Zn_4_ species. The spectrum for MT4b at pH 4.2 is broadly similar to that of MT4a at pH 4.4, although Zn_4_ and Zn_5_ species were still more prominent for MT4b. Finally, at even lower pH (< 3.5), the apo forms were the major species for both proteins, but in both cases, Zn_1_ and Zn_2_ species were still observed, albeit at low abundances.

In summary, both MTs produced a range of under-metallated species during a pH titration, but at pH values around the pH(1/2), MT4b displayed a higher degree of metallation, even though in both examples shown in Fig. 3, the pH of the MT4b solutions was slightly lower than those of the corresponding MT4a solutions.

**Fig. 4:**
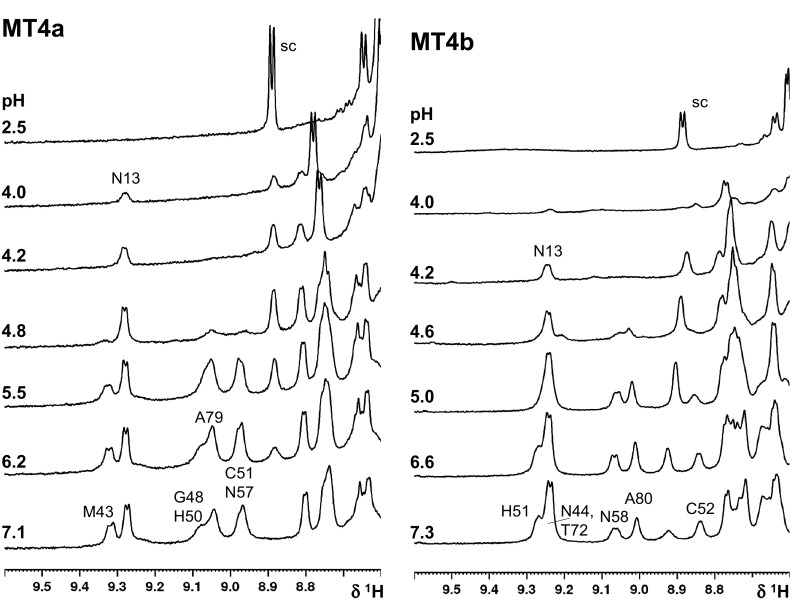
Low-field region of selected NMR spectra of MT4a and MT4b at different pH values (400 µM protein, 50 mM Tris-D_11_, 10% D_2_O, 50 mM NaCl, 298 K). The backbone amide proton resonances for representative residues are annotated. For both proteins, domain II residues are affected at higher pH values than those of domain I

Subsequently, 1D ^1^H NMR spectroscopy was employed to investigate the consequences of proton-induced zinc(II) loss on protein folding. As expected for MTs [80], the apo proteins present at pH 2.5 are essentially unfolded, as judged [92] by the complete loss of chemical shift dispersion (Supplementary Fig. S1). Figure 4 focuses on the low-field region which at neutral pH harbours relatively well-resolved backbone NH resonances from both domains. These ^1^H NMR data confirm that the most significant changes occur between pH 5 and 4, as was also observed by UV spectroscopy [36] and ESI-MS (Fig. 3), thus demonstrating that metal loss (UV and ESI-MS) and protein unfolding (NMR) are correlated. Most importantly, the data suggest that for both proteins, the two domains have different pH stabilities. Proton-driven demetallation first leads to unfolding of domain II, followed by unfolding of domain I at lower pH—for example, the MT4a domain I Asn13 resonance shows nearly undiminished intensity at pH 4.8, whilst the various domain II resonances (Met43, His50, Asn57, Gly58, and Ala79) are greatly reduced in intensity. The situation for MT4b is similar, with the resonances for Asn58 and Ala80 being left just about discernible at the somewhat lower pH of 4.6. At pH 4.2, domain II resonances have disappeared for both proteins, whilst the Asn13 resonance from domain I has reduced in intensity, but is still clearly present. The ^1^H NMR data do not allow discerning any differences between different sections within the domains for either protein, nor were any obvious differences in the behaviour of the two proteins evident. It is hence not possible to correlate the differences in speciation seen by ESI-MS with differences in protein folding. Most importantly, however, combining the ^1^H NMR with the ESI-MS data allows inferring that the most pH-labile zinc(II) ions in both homologues reside in domain II.

### Reaction with EDTA: UV–visible spectroscopy

Inspired by suggestions that type 4 MTs may function as zinc(II) donors to nascent Zn-requiring proteins during or after germination [39, 55], we studied the zinc(II) transfer kinetics of the two *A. thaliana* MT homologues to the metal chelator EDTA, a commonly used probe to study the lability of MT-bound metals [80, 93–97].

**Fig. 5:**
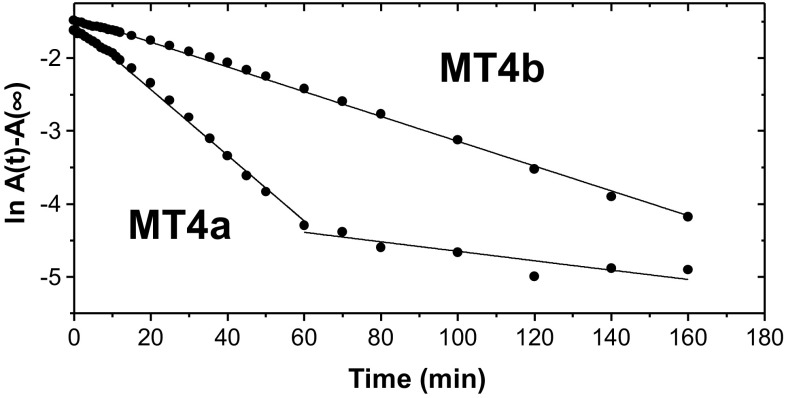
Semi-logarithmic plot of the reaction of Zn_6_MT4a and Zn_6_MT4b with a 100-fold excess of EDTA at pH 7.4 (5 µM protein, 25 mM Tris buffer, pH 7.33, 298 K). The lines correspond to linear fits, from which rate constants (*k*_obs_) for pseudo-first-order reaction kinetics were derived

Zinc transfer to EDTA under pseudo-first-order conditions (100-fold molar excess) was monitored as a decrease in absorbance of Zn–S bonds at 220 nm using UV–visible spectroscopy. Although Tris–Cl does not buffer well at pH 7.3, this buffer system was chosen for both UV–Vis and NMR studies, as many related studies on MTs had also been carried out in Tris buffer, e.g., [95–97]. The pseudo-first-order plots in Fig. 5 show that MT4a initially transferred zinc(II) faster than MT4b. Under these conditions, only one phase was observed for MT4b, whilst MT4a displayed biphasic kinetics. The pseudo-first-order rate constant for MT4b was *k*_obs_ = 2.8 × 10^−4^ s^−1^. The faster step for MT4a proceeded with *k*_obs_ = 7.5 × 10^−4^ s^−1^; the slower step with *k*_obs_ = 9.3 × 10^−5^ s^−1^. Hence, the initial metal release from MT4a was almost three times faster than for MT4b under these conditions. MT4a also reacted faster than MT4b when lower EDTA:protein ratios were employed, with clearly decreased reaction rates for either protein (Supplementary Fig. S2). Importantly, the latter fact indicates that the reaction rate depends on the EDTA concentration, i.e., the rate-determining step is (at least) bimolecular and involves direct interaction between the protein and the chelator. Thus, the two type 4 MTs behave in a broadly similar way to MTs that have been studied by this method [93, 95, 96], but with some subtle differences to wheat E_C_ [93] as well as to each other. The significance of the observations from UV spectrophotometry will be further discussed once MS and NMR results have been introduced.

### Reaction with EDTA: native ESI-MS and NMR spectroscopy

**Fig. 6:**
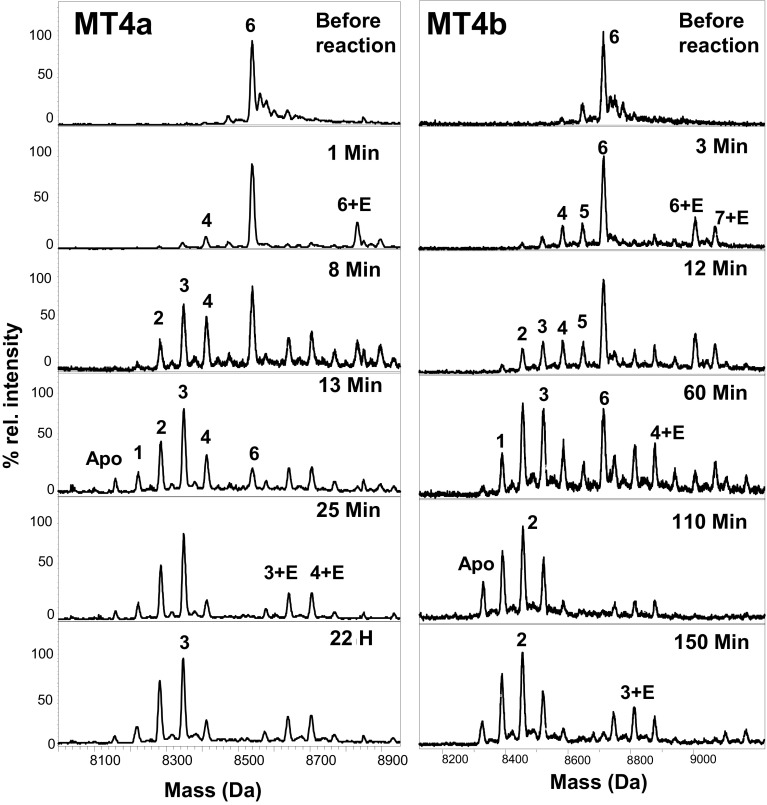
Representative deconvoluted mass spectra taken at different time points during the reaction of Zn_6_MT4a or Zn_6_MT4b (270 µM protein, 10 mM NH_4_HCO_3_ buffer, pH 7.8, 298 K) with equimolar EDTA (1.62 mM). Data were acquired on diluted aliquots (25 µM, 10% v/v CH_3_OH, 10 mM NH_4_HCO_3_ pH 7.8)

**Fig. 7:**
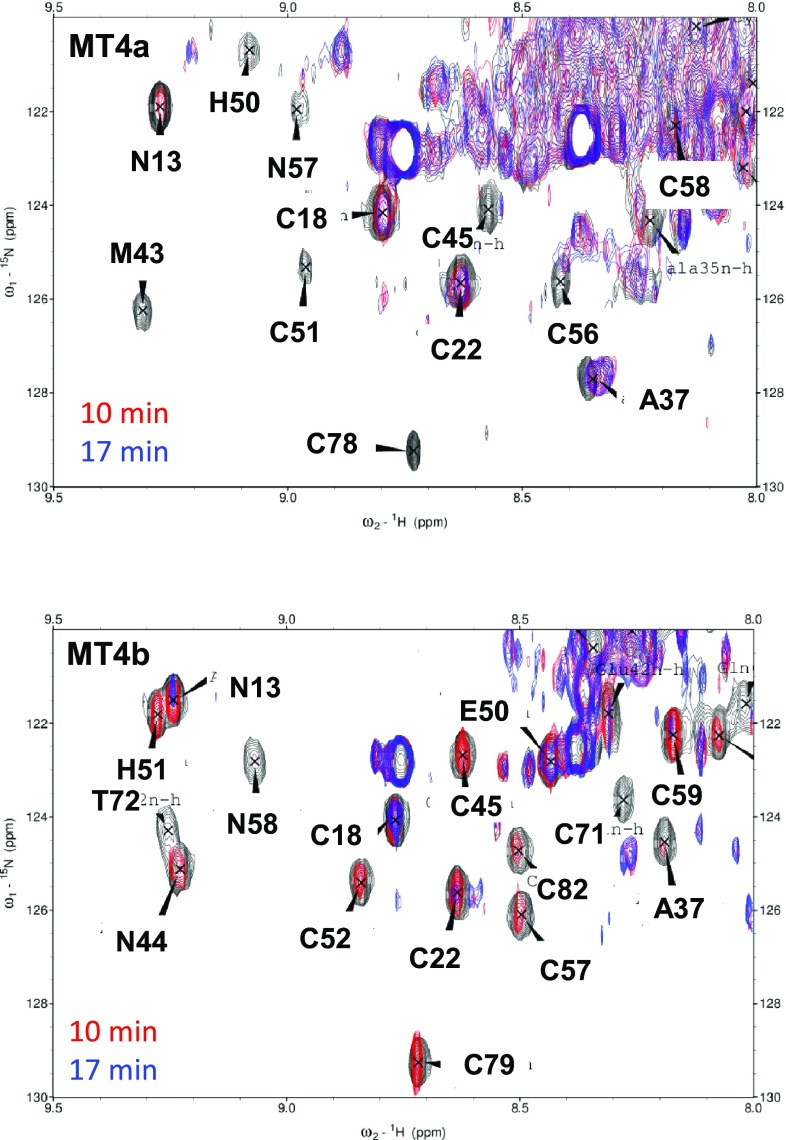
Representative section of overlaid 2D [^1^H, ^15^N] HSQC spectra during the reaction of ^15^N-labelled Zn_6_MT4a (251 µM) or Zn_6_MT4b (267 µM) with equimolar (with respect to [Zn]) EDTA (50 mM Tris-D_11_, 50 mM NaCl, 10% D_2_O, pH 7.4, 298 K). Spectra before the reaction are shown in black, spectra after 10 min in red, and spectra after 17 min in blue. The immediate disappearance of domain II resonances for MT4a is evident, whilst domain I resonances persist for both proteins during the first 17 min, albeit at weakened intensities. The very strong persistent peaks are due to a contaminating peptide in both cases

Native ESI-MS has been used previously to monitor the speciation of MTs during their interactions with other metal acceptors including proteins [93, 96–101]. Here, we have combined this technique with 2D [^1^H, ^15^N] HSQC NMR spectroscopy to correlate the folding state of individual domains in different metalloforms. By necessity, the reaction conditions differ from those employed for UV spectrophotometry, because NMR spectroscopy requires higher protein concentrations. Hence, all MS and NMR timecourse experiments were carried out with 250–280 µM solutions of proteins, but only equimolar amounts of EDTA with respect to [Zn^2+^], to bring the reaction times within a manageable range (Figs. 6, 7). We also note that different buffer conditions and ionic strengths were used for ESI-MS and NMR experiments; therefore, reaction times for these two experiments cannot be directly compared. Broadly speaking, it appears that the reactions under ESI-MS conditions (10 mM NH_4_HCO_3_ pH 7.8, *I* = 10 mM) proceeded more slowly than those observed by NMR (50 mM Tris, 50 mM NaCl, pH 7.4, *I* = 54 mM).

Mass spectra (Fig. 6) recorded immediately after mixing reveal the formation of EDTA adducts for both proteins, in accordance with the conclusion from UV–Vis spectroscopy, i.e., that the reaction requires formation of a complex between protein and chelator. Interestingly, such complexes were even present at the end of the reaction, where they were observed for several under-metallated species. EDTA adducts formation has been previously observed for bacterial SmtA [96] and wheat E_C_ [93], and also has been postulated to occur during reaction with mammalian MTs [95]. In addition, the requirement for direct interaction between MT and recipient proteins to enable metal transfer has been highlighted previously [102].

In either case, the reaction did not lead to the complete demetallation at the chosen EDTA ratio, a finding in accordance with the previous work on wheat E_C_ [93]. Inspection of the entire timecourse indicates that the reaction with MT4a was essentially completed after 25 min, whilst the period required to reach equilibrium was, under these conditions, at least four times longer for MT4b. Thus, metal ions are lost much faster from Zn_6_MT4a than from Zn_6_MT4b also when EDTA is provided in stoichiometric amounts. Comparison of the mass spectra at the 12/13 min time point further corroborates this conclusion: the Zn_6_ species was still dominating MT4b speciation, along with low intensity peaks for Zn_5_, Zn_4_, Zn_3_, Zn_2_, and Zn_1_ species, whilst the most abundant species for MT4a was already Zn_3_, with the Zn_6_ species reduced to < 10% relative abundance. Furthermore, the speciation for the proteins differed during as well as at the end of the reactions. One salient feature is the almost complete absence of a Zn_5_ species for MT4a, whilst this was clearly observable for MT4b during the first 60 min of the reaction. Another important observation is the dominance of the Zn_3_MT4a species throughout the reaction—including after equilibration for 22 h. In contrast, the most abundant species for MT4b at this time point was the Zn_2_ species, although the Zn_3_ species was the second-most abundant metalloform. In addition, although the MT4a apo form was observed after 13 min already, its relative abundance remained well below 10% (Fig. 6). The MT4b apo form first appeared at 60 min, but also remained at low relative abundance.

The most important conclusion from the ESI-MS experiments is that metal ions are lost in a fairly non-cooperative fashion from MT4b, with no evidence for any “cluster collapse” after removal of the first Zn ion: Cooperative metal loss is expected to lead to greatly diminished abundance of species corresponding to partially loaded domains [82–85]; therefore, a very different pattern of abundances for the various under-metallated species during the reaction may be expected if cooperativity was in effect. In contrast, the peculiar absence of the Zn_5_ species in MT4a indicates that loss of the first Zn ion facilitates loss of a second one, leading to a brief build-up of the Zn_4_ species, which, however, also reacts fairly rapidly to give the Zn_3_ species.

To shed some light on the likely locations of the most reactive Zn sites and remaining Zn ions, and whether the two domains in each protein displayed different reactivities that may help explain the observations from UV–Vis spectroscopy and ESI-MS, 1D ^1^H (Supplementary Fig. S3) and 2D [^1^H, ^15^N] HSQC NMR spectroscopy (Fig. 7) was employed. 2D [^1^H, ^15^N] HSQC spectroscopy has the advantage of eliminating spectral overlap, enhancing the ability to extract residue-specific information throughout the fingerprint region, whereas 1D ^1^H NMR spectroscopy allows faster sampling of time points, and also yields information on relevant side-chain protons, for example the aromatic protons of His residues. Moreover, 1D spectra also contain quantitative information on the ratio between free and Zn-bound EDTA. Both 1D and 2D data sets reveal that metal transfer to EDTA is accompanied by the disappearance of resolved backbone NH peaks, consistent with the unfolding of the proteins. The higher initial reactivity of Zn_6_MT4a compared to Zn_6_MT4b was also evident from both data sets (Fig. 7 and Supplementary Fig. 3), especially regarding the very first time points in each case (ca. 5 min for the 1D data, and ca. 10 min for the 2D data). For both proteins, the presence of substantial amounts of free EDTA at the end of the reactions monitored by 1D ^1^H NMR (Supplementary Fig. S3) confirmed that equimolar EDTA was insufficient for complete demetallation.

The 2D NMR data show that for both proteins, domain I was initially less reactive than domain II, as in both cases, domain I residues were visible at the 10 and 17 min time points—whereas this was not the case for domain II (see below). In fact, a proportion of the domain I resonances persisted throughout the reaction for both proteins, albeit at clearly diminished intensities (Supplementary Fig. S4). The decreasing intensities for both domains indicate that in principle, both domains of both proteins are able to react with EDTA simultaneously, albeit at different rates—at least when EDTA is supplied in equimolar amounts. Interestingly, the intensity of the domain I resonances for MT4a even increased slightly after the 25 min time point, perhaps an indication for slow metal redistribution after the initial removal from domain I. This observation highlights that the kinetic product(s) of the reaction with EDTA may not necessarily be identical to the thermodynamic products. It is somewhat surprising that this redistribution/refolding seems to be a slow process, as it has been pointed out that MT metallospecies, due to fast on/off rates, usually equilibrate within milliseconds with each other [60]. Either way, the persistence of domain I resonances—only observed in the more sensitive 2D HSQC experiment—suggests that for both proteins, at least some of the Zn_2_ (and Zn_3_) species observed by ESI-MS correspond to fully metallated domain I.

Significant differences between the two homologues were observed for domain II. For MT4a, all domain II resonances had already been lost after 10 min (Fig. 7a). A corresponding 1D data set indicates that ca. 3.8 equivalents of EDTA had reacted by this time already (Supplementary Fig. S3). In contrast, the 2D MT4b data show resolved domain II resonances at the same 10 min point. It is noteworthy that all four ligand residues that form the mononuclear site (His43, His51, Cys57, and Cys59) were still clearly resolved. The residues maintaining ordered structure also included all remaining four Cys residues in the second Cys-rich stretch of the MT4b sequence (Cys46, Cys48, Cys52, and Cys54). The previous work on wheat E_C_ mutant proteins had indicated that the Zn-bound status of the equivalent of His51 was critical to structuring the entire domain II via crosstalk to these four residues [61]. Residues Cys77, Cys79, and Cys82 were still present as well. The fact that resonances for the mononuclear site of domain II remained visible in the initial stages, together with the disappearance of the resonances for Cys71 and 73, may be taken to suggest that the initial zinc(II) release from Zn_6_MT4b occurred not from the mononuclear site, but preferentially from the three-metal cluster, although we would caution that resonances for the three residues Cys71, Thr72, and Cys73 were already weaker in the unreacted protein.

In summary, the likely origin of the different metal transfer dynamics of MT4a and MT4b resides predominantly in domain II, with MT4a showing more rapid and more cooperative metal transfer to EDTA. In the following section, we will seek to identify the cause for this difference.

## Discussion

Studying metallospecies and protein folding as affected by proton-driven demetallation has revealed that for both MT4a and MT4b, domain II is more pH-labile than domain I. A re-analysis of published data for wheat E_C_ [45] indicates that this is also the case for the latter protein. E_C_ produced a prominent Zn_4_ species at moderately acidic pH that coexisted with either the Zn_6_ species (pH 5.5) or the Zn_2_ and apo species (pH 4.0), whilst Zn_5_, Zn_3_ or Zn_1_ were much less abundant; yet, the loss of two zinc(II) ions from Zn_6_E_C_ did not correspond to cooperative demetallation of the Zn_2_ domain I as might have been concluded, as the NMR signals from this domain persisted at lower pH than those for domain II [103]. Therefore, neither wheat E_C_ nor *A. thaliana* MT4b lose their domain II-bound zinc(II) ions cooperatively as far as proton-driven metal loss is concerned. There is some indication for moderately cooperative loss of zinc(II) from domain II of MT4a, where the Zn_2_ species that most likely corresponds to fully metallated domain I was most abundant at pH 4.9. Furthermore, since the backbone amide ^1^H NMR signals for the Zn_6_ forms of either protein neither shift nor broaden, the species observed are unlikely to be in chemical exchange with other species. Hence, the decreases in intensity correspond to the decrease in the concentration of the fully metallated domains in both cases. This also means that the Zn_4_ or Zn_5_ species for MT4b, which are present at significant amounts above the pH(1/2), do not seem to give rise to new dispersed NMR signals. Hence, partially metallated domains are likely to be structurally disordered.

Regarding metal transfer dynamics, we will first attempt to contextualise and integrate the results from the three techniques employed to monitor the reactions with EDTA. The UV–Vis timecourse data revealed monophasic reaction kinetics for MT4b, and biphasic kinetics for MT4a, with the first phase of the latter being considerably faster than the single phase for MT4b. For full-length mammalian MTs, different phases were ascribed to differences in the reactivity of their two domains [31, 93, 95]. Zn_6_E_C_ from wheat appeared to display triphasic reaction kinetics, although the fastest step was only discernible at lower EDTA:protein ratios, and could hence not be quantified under appropriate pseudo-first-order conditions [96]. This fastest step was suggested to correspond to the loss of up to 3 Zn ions, followed by a second slower phase with a rate of ca. 5 × 10^−4^ s^−1^, between the rates for the fastest observed step for MT4a and for the only step seen for MT4b. In the case of wheat E_C_, it was not possible to determine whether the most reactive Zn ions originated from domain I or II. The slowest third phase observed for wheat E_C_ was attributed to protein degradation. This did not appear to be a major contribution within the timeframe of the present experiments. Furthermore, monitoring the reaction at lower EDTA:protein ratios for MT4a or MT4b gave no clear indication of whether a faster step for either MT4a or MT4b exists (Supplementary Fig. S2), but comparison of the time required to completely deplete the Zn_6_ form (25 min for MT4a as well as for wheat E_C_, 70 min for MT4b) suggests that Zn_6_MT4b reacted considerably more slowly than either wheat Zn_6_E_C_ or Zn_6_MT4a; thus, it is most likely that the fast component observed for wheat E_C_ does not occur for MT4b.

In the case of mammalian MTs, kinetic data were compatible with removal of the first metal ion being the rate-determining step for the reactivity of the entire cluster, with the loss of the subsequent cluster ions being much faster than the first—in essence, metal loss was cooperative within the individual domains [31, 94]. In contrast, our native ESI-MS data indicate that zinc(II) loss from MT4b is largely non-cooperative, as the full range of species was observed with detectable and comparable intensities (Fig. 6). For MT4a, the absence of the Zn_5_MT4a species points to a degree of cooperativity—but the NMR results indicate that the observed Zn_4_ species do not correspond to fully loaded domain II, but instead include species with fully loaded domain I. This also means that these Zn_4_ species harbour domain II with two Zn ions bound—reminiscent of the Zn_4_ species observed for wheat E_C_ at moderately acidic pH. Although these under-metallated species have been generated in different ways, it is clear that the three cluster sites are not equivalent in either MT4a or wheat E_C_—irrespective of at which sites the remaining two ions are bound. In any case, the clear occurrence of the Zn_4_MT4a species indicates that removal of the first ion did not lead to “cluster collapse” for MT4a either, although it is clear that the four zinc(II) ions bound to domain II are more prone to transfer than the two in domain I.

Finally, the NMR results clearly indicate that in both proteins, both domains are capable of metal transfer, a conclusion that was also reached for wheat E_C_ [97]. Thus, in the case of plant MTs, the simple kinetics displayed may not always be a consequence of single rates being observed for each domain, but of similar rates for several different sites in different metallated species. Thus, it may be concluded that the initial reactivities of the two domains in MT4b are more similar to each other, and hence, any small differences in reactivity are not resolved at low protein concentration and 100-fold excess of EDTA. In turn, the biphasic kinetics observed for MT4a may be a reflection of the distinct reactivities of the two domains, as observed by NMR spectroscopy, with the fast phase dominated by domain II losing 3–4 zinc(II) ions. Thus, the most salient finding from integrating UV–Vis and NMR results is the comparatively high reactivity of domain II of Zn_6_MT4a.

The differences observed between MT4a and MT4b must ultimately arise from differences in their sequences (as highlighted in Fig. 2c), with consequences for their 3D structures and protein dynamics. Our NMR spectroscopic studies suggested that there were no large differences in the reactivity of the two domains I, but that domain II is responsible for the most significant differences in reactivity. Therefore, this discussion will largely focus on the latter domain, but will also include a brief inspection of the two domains I.

Structural effects of differences in the primary structures are reflected in the chemical shift comparison shown in Fig. 2c. It is evident that the largest differences in backbone shifts are observed where residues are not conserved. For these particular residues (highlighted in red), this is, in the first instance, a consequence of their different chemistry that impacts on their random-coil chemical shifts, and hence also on their chemical shifts in a folded protein. This is particularly obvious where one of the proteins harbours a glycine residue, as Gly backbone amide ^15^N chemical shifts are very different to those of most other amino acids. To a certain degree, differences in a given position will also impact on the directly adjacent residues. Therefore, the seemingly large chemical shift differences for some of the red residues and their neighbours are not remarkable per se, but can be used as a visual aid to pinpoint potential areas of interest.

**Fig. 8:**
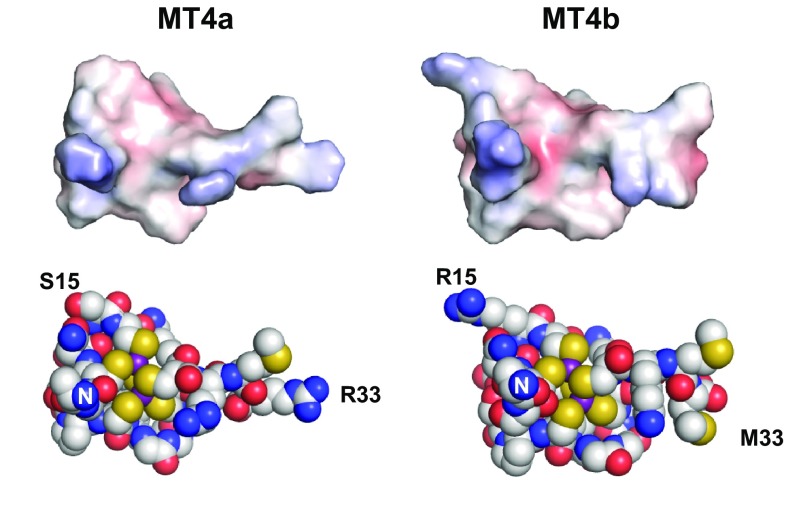
Comparison of electrostatic potential surfaces and space-fill models of domain I. The models are oriented to show the zinc(II) ions from the most accessible side. The long presumably disordered N-terminal tails have been removed, with the first residue depicted being Ala10 in both cases. This has led to an artificial positive charge at the new N-terminal residue; since the main purpose of this analysis was the comparison between MT4a and MT4b, we have refrained from introducing an artificial N-terminal acyl cap. The slight increase in negative charge in the vicinity of the metal cluster in MT4b originates from differences in the orientation of the backbone carbonyl oxygens of residues G11/S11; this is a consequence of the modelling process and should not be over-interpreted. Residues N26 and E26 are at the back of the molecules. Neither the S15/R15, nor the N26/E26, or indeed the M33/R33 substitutions have a major impact on electrostatics around the zinc(II) sites. The surfaces are coloured by electrostatic potential as calculated by APBS, using ± 5.0 as maximal negative/positive potential, respectively. Carbon, oxygen, and nitrogen atoms are labelled in CPK mode, with Zn ions in purple and sulfur atoms in yellow

Within domain I, two potentially important substitutions concern S15/R15 and N26/E26 (the latter residues are highlighted in grey as they could not be assigned for either protein). Taken together, these two substitutions do not alter the overall charge of the domain. To discern whether one might expect an effect of these substitutions on the immediate environment of the zinc(II) ions, we have generated homology models of the two domains I, based on the published structure of the wheat E_C_ γ domain [58]. Inspection of the surface of the side from which the two zinc(II) ions are accessible reveals no major differences in surface potential near the metal ions (Fig. 8), in broad agreement with our experimental observations.

**Fig. 9:**
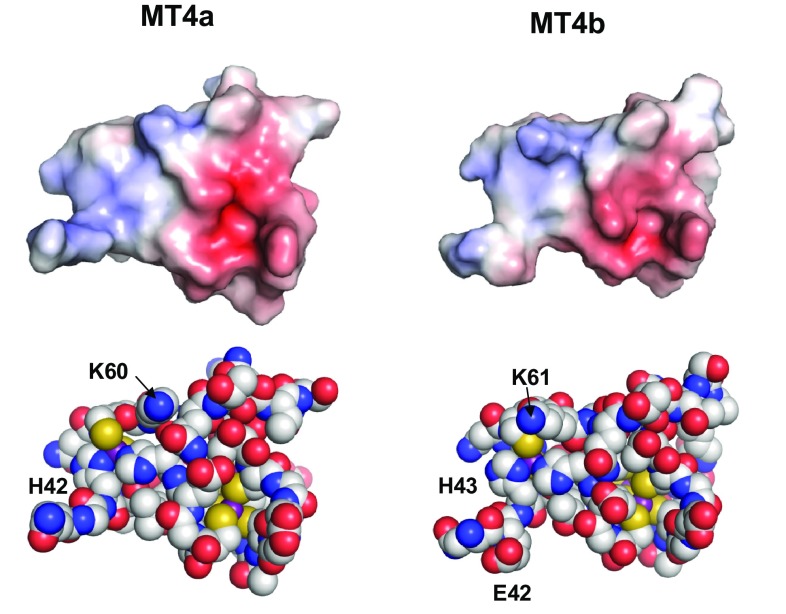
Comparison of electrostatic surfaces and space-fill models of domain II. The models are oriented to show the mononuclear site (indicated by the H42/H43 labels) from its most accessible side. In contrast to the Zn_3_Cys_9_ cluster, around which there is an accumulation of negative charge, the mononuclear site is characterised by positive surface potential which may facilitate nucleophilic attack as well as complex formation with a negatively charged partner molecule. The models suggest that besides the zinc(II) ion, K60/61 also contributes to positive charge, and that the reduction in positive potential in MT4b is attributable to E42. The surfaces are coloured by electrostatic potential as calculated by APBS, using ± 5.0 as maximal negative/positive potential, respectively. Carbon, oxygen and nitrogen atoms are labelled in CPK mode, with Zn ions in purple and sulfur atoms in yellow

Within domain II, the most remarkable dissimilarities occur around the first histidine residue (His42/His43). MT4a harbours a GHMV (41–44) motif, whilst for MT4b, the sequence is EHNT (42–45). Indeed, many type 4 MTs have the equivalent of Glu42 (Fig. 1). It can be anticipated that a negative charge may somewhat screen the mononuclear site against attack by nucleophiles—i.e., any reagent with a negative (partial) charge, which is of course also a property for any metal ligand including EDTA and potentially zinc(II) acceptor proteins. To examine the plausibility of this hypothesis, homology models of domain II of MT4a and MT4b have been generated based on the published structure of the beta-E domain of wheat E_C_ [58]. In both MT4a and MT4b, the mononuclear site is characterised by a slightly positive surface potential, especially when the most accessible side of the site is considered (Fig. 9). Besides the zinc(II) ion itself, another contributor to positive charge in this region is the fairly well-conserved Lys60/61. Although the differences appear to be subtle, it is evident that the mononuclear site in MT4a is slightly more positive than that in MT4b. The reduced positive charge in MT4b is likely a consequence of longer range effects of Glu42. We propose that more positive charge around a zinc(II) site may facilitate approach of the negatively charged carboxylate groups of EDTA, and hence, the absence of the equivalent of Glu42 is likely to render the mononuclear zinc(II) ion in MT4a more reactive than that in MT4b. The electrostatic surface images also suggest that the Zn_3_Cys_9_ clusters are surrounded by substantial negative charge. This is mostly a consequence of the formal − 3 charge of such clusters, which is not fully compensated by the presence of the two lysine residues in this domain. It should be noted that the side-chain conformations of lysine residues in our models are variable, and in solution would be quite flexible as well. It is, therefore, possible that their positive charges are at least occasionally closer to the negatively charged clusters in the vicinity, and this may in principle reduce average negative charge in certain areas to some degree, and thus facilitate nucleophilic attack.

Comparing electrostatics for all likely points of EDTA binding and nucleophilic attack (Figs. 8, 9), the mononuclear site in MT4a is the most positively charged, in agreement with our combined observations from UV–Vis, ESI-MS, and NMR spectroscopy. The next reactive site appears to be the mononuclear site in MT4b, also in broad agreement with our data.

The above considerations are based on the hypothesis that bimolecular assemblies are required for efficient metal transfer. Indeed, such assemblies were observed in this work by ESI-MS, and electrostatics are undoubtedly important for understanding the likelihood for their formation. However, another critical contributor to metal lability in MTs is protein dynamics. Although we have not carried out extensive NMR-based backbone dynamics measurements, the HSQC spectra shown in Figs. 2a, b and 7, in conjunction with our sequential assignments (Fig. 2c), allow some pertinent qualitative observations. The HSQC spectra of MT4a (Figs. 2a, 7) were consistently characterised by a larger amount of unresolved intensity in the random-coil region in the middle of the spectra, and numerous domain II residues gave rise to comparatively weak signals, whilst this was not the case for MT4a domain I resonances. This strongly suggests that domain II of MT4a is inherently more flexible than domain II of MT4b. One consequence of this increased flexibility is highlighted by the grey bars and letters in Fig. 2c. The corresponding residues are those that could not be assigned for MT4a, but for MT4b. The difficulties in identifying the respective cross-peaks in the MT4a data indicate that either the chemical environment, or the backbone dynamics, or, indeed, both are substantially different between MT4a and MT4b, as otherwise, there should have been an unassigned peak in the MT4a HSQC spectrum with similar shifts to an assigned peak in the MT4b spectrum. For Cys72 (MT4a), this can be ruled out most definitively (due to the extreme low-field shift (9.85 ppm) of the Cys73 proton in MT4b). This means that structure and/or dynamics of this particular stretch differ significantly between the two proteins. We also note that the ^1^H resonance for Cys73 in MT4b is quite weak and broad; so in MT4b, this region is characterised by higher flexibility than the remainder of domain II. It is possible that the same is true for this region in MT4a, which coupled with an inherently higher flexibility of the entire domain, may lead to dynamics on the intermediate exchange regime for Cys72 in MT4a. It is noteworthy that the primary sequences in this stretch are identical; therefore, differences observed here must be a consequence of changes elsewhere in the protein sequence. It is fairly unlikely that the conservative Ser80/Thr81 or Thr83/Ala84 substitutions would have such a remarkable effect on backbone dynamics elsewhere in the domain; the more likely suspects are again the substitutions around His42/43. Asn44 and Thr45 in MT4b are both hydrophilic residues capable of engaging in hydrogen bonding; indeed, in our model, the amide side chain of Asn44 has an H-bond with that of Asn55 (not shown). In contrast, such stabilising H-bonds are not possible for Met43 or Val44. These two residues together form a small hydrophobic patch on the protein surface that is rather unusual for an MT. Whilst the interaction between these two residues may be mutually stabilising, hydrophobic sidechains on a protein surface are inherently unfavourable, and thus, we hypothesise that these two residues are significant contributors to the higher structural flexibility of domain II in MT4a. Such increased flexibility is likely to increase the lability of bound metal ions, and this may be a further contribution to the faster metal transfer dynamics of MT4a—and this may not only affect transfer from the mononuclear site, but also from the Zn_3_Cys_9_ cluster.

## Conclusions

The suggested biological function of type 4 MTs includes zinc(II) donation to zinc(II)-requiring proteins, peptides, or small molecules during seed maturation and/or germination [37, 50, 55]. This concept is supported by in planta studies in *A. thaliana* that demonstrated MT4 protein localisation in the embryo [55]. More recently, type 4 MT protein was also detected in the aleurone layer of maturing and mature seeds of the monocotyledonous plant barley [52, 104]. This tissue is important for nutrient transport and seed filling. Taken together, these in vivo studies strongly support a role of type 4 MTs in supplying zinc(II) to nascent zinc(II)-requiring proteins, during embryogenesis, seed maturation, and/or during germination. It is, therefore, of general importance to investigate their ability to transfer zinc(II) to suitable acceptor molecules. Our studies using EDTA as a model zinc(II) acceptor confirm that *A. thaliana* MT4a and MT4b principally fulfil essential criteria to act as cellular zinc(II) donors: at least some of their bound metals are bound in a fairly labile fashion, allowing zinc(II) transfer to EDTA to occur with rates comparable to those observed for other MTs. We note that another important aspect in this scenario is metal affinity, and that in the case of wheat E_C_, it has been found that the average apparent binding constant for zinc(II) was particularly low at higher ionic strength (log*K* = 8.6 at pH 7.4 and *I* = 105 mM) [45]. It is conceivable that such low affinity might favour metal transfer from the MT.

All our experiments started with fully metallated MTs. It may, however, be noted that there is increasing evidence that many MTs are only partially metallated in vivo. The prevalence of partially metallated MTs and their relevance in the cellular context has been pointed out repeatedly for mammalian MTs [29–31, 60, 84, 105, 106], but there are also several reports where under-metallated plant MT4 s have been isolated [52, 107]. Indeed, the stoichiometries found for sesame and barley were approximately 2–3 zinc(II) per protein, and based on our observations by ESI-MS and the high level of conservation in this domain (Fig. 1), it is tempting to speculate that these species might harbour fully loaded domains I. In the case of wheat E_C_ [45, 61] and the two homologues from *A. thaliana,* these are characterised by high thermodynamic stability, as judged from both pH-dependent studies (Figs. 3, 4) and analysis of the end products of the EDTA reactions (Figs. 6, 7).

However, the most intriguing finding of the current study concerns the differences observed between two closely similar homologues that both occur in the seeds of a single plant. In planta observations had suggested that *A. thaliana* MT4a and MT4b work cooperatively where the role of MT4a is to distribute zinc(II) via the vasculature and MT4b functions as a store for zinc(II) throughout the embryo [55]. Although no differences in zinc(II)-binding strength were evident as judged form the pH(1/2) values determined earlier [37], the faster kinetics for zinc(II) transfer from MT4a and the slower kinetics for MT4b align well with these suggestions. In particular, our NMR studies, coupled with inspection of homology models and their electrostatic surfaces, have identified the mononuclear Cys_2_His_2_ site and its surroundings as the lynchpin that governs the reactivity of these two type 4 MTs, with non-conservative substitutions around the first histidine residue playing a crucial role for electrostatics and overall backbone dynamics.

Ultimately, it is clear that also zinc(II) bound to MT4b will need to be mobilised; this may or may not occur through direct transfer to other proteins. Several other options for zinc(II) release from MTs exist; these include oxidative reactions with electrophiles such as peroxide or oxidised glutathione [80], but may also encompass proteolytic degradation of the protein, as shown for wheat E_C_ [48]. In addition, changes in pH or ionic strength may also modulate zinc(II) release. Within this framework, it can be envisaged that the two MTs may transfer or release their cargo at different times, different locations, and perhaps to different acceptors. Given their different reactivities, it is also possible that in each case, the two domains may serve different purposes.

## Electronic supplementary material

Below is the link to the electronic supplementary material.

Supplementary material 1 (PDF 768 kb)

## Abbreviations

EDTA: Ethylene-diamine-tetraacetic acid
ESI-MS: Electrospray ionisation mass spectrometry
HSQC: Heteronuclear single quantum coherence
ICP-OES: Inductively coupled plasma-optical emission spectroscopy
MT: Metallothionein
NMR: Nuclear magnetic resonance
NOESY: Nuclear Overhauser enhancement spectroscopy
pMT: Plant metallothionein
TOCSY: Total correlation spectroscopy

## Footnotes

1 Note that residue numbering in the literature is not unanimous; the numbering used here is based on the full-length sequence of natively isolated wheat E_C_-I/II in which the N-terminal methionine is cleaved, i.e., the first residue is Gly1 [45].

2 In the MT literature, it is common to use the term “isoform” to refer to different MT proteins of a particular organism. However, this is strictly speaking incorrect, as “isoforms” are derived from the same gene, e.g. by alternative splicing. We, therefore, prefer the term “homologue”.
