# Supplementary material for: Differential reactivity of closely related zinc(II)-binding metallothioneins from the plant Arabidopsis thaliana
Source: J Biol Inorg Chem. 2017 Dec 7;23(1):137–54. doi: 10.1007/s00775-017-1516-6 (PMC5756572; doi:10.1007/s00775-017-1516-6)
Supplement: Supplementary file 1 — Supplementary material 1 (PDF 768 kb) [file 775_2017_1516_MOESM1_ESM.pdf]

Differential reactivity of closely related zinc-binding metallothioneins from the plant *Arabidopsis thaliana*

Hasan T. Imam and Claudia A. Blindauer

Supplementary Material

Table S1. ^{1}H and ^{15}N backbone amide chemical shifts for *A. thaliana* MT4a and MT4b

Figure S1. Full 1D ^{1}H NMR spectra of MT4a and MT4b at pH 2.5

Figure S2. Timecourse data for the reaction of MT4a and MT4b with different excesses of EDTA

Figure S3. Selected regions of 1D ^{1}H NMR spectra, showing increasing formation of the Zn-EDTA complex concomitant with protein unfolding

Figure S4. [^{1}H,^{15}N] HSQC spectrum of MT4a after overnight reaction with equimolar EDTA

Table S1. ^{1}H and ^{15}N backbone amide chemical shifts for *A. thaliana* MT4a and MT4b

MT4a	H	N	MT4b	H	N
Cys12	8.124	120.2	Cys12	8.073	122.2
Asn13	9.273	121.9	Asn13	9.241	121.4
			Asp14	8.222	119.9
Ser15	8.544	112.3	Arg15	8.457	116.5
Cys16	7.602	121.1	Cys16	7.535	119.2
Gly17	8.745	112.1	Gly17	8.724	111.9
Cys18	8.798	124.2	Cys18	8.764	124
Pro19			Pro19		
Ser20	8.298	115.0	Ser20	8.289	114.9
Pro21			Pro21		
Cys22	8.631	125.6	Cys22	8.631	125.6
Pro23			Pro23		
Gly24	8.652	110.8	Gly24	8.643	110.7
Gly25	8.477	107.3	Gly25	8.393	107
Asn26			Glu26		
Ser27	7.775	112.2	Ser27	7.777	112.1
Cys28	7.216	122.7	Cys28	7.275	122.7
Arg29	8.216	132.5	Arg29	8.168	132.5
Cys30	8.021	122.0	Cys30	7.953	121.7
Arg31	7.756	121.2	Lys31	7.813	121.6
Met32	8.053	119.3	Met32	8.088	119.4
Arg33			Met33		
			Ser34		
Glu34			Glu35		
Ala35	8.225	124.3	Ala36	8.186	124.5
Ser36	8.340	114.9	Ser37	8.251	114.9
Ala37	8.360	127.7	Gly38	8.396	110.6
Gly38	8.233	108.2	Gly39	8.276	108.7
Asp39			Asp40	8.339	120.2
Gln40			Gln41	8.215	119.4
Gly41	8.387	109.0	Glu42	8.307	121.7
His42	8.124	117.2	His43	8.559	119.1
Met43	9.312	126.3	Asn44	9.23	125.1
Val44	7.972	112.6	Thr45	8.48	111.2
Cys45	8.572	124.0	Cys46	8.621	122.6
Pro46			Pro47		
Cys47	7.382	115.6	Cys48	7.37	116.4
Gly48	9.049	112.8	Gly49	8.667	111.9
Glu49	8.401	122.7	Glu50	8.432	122.7
His50	9.082	120.7	His51	9.272	121.7
Cys51	8.959	125.3	Cys52	8.839	125.3
Gly52	8.379	113.3	Gly53	8.368	113.2
Cys53	7.465	118.8	Cys54	7.439	118.5
Asn54	7.065	119.4	Asn55	7.027	118.7
Pro55			Pro56		

Cys56	8.417	125.6	Cys57	8.497	126
Asn57	8.974	122.0	Asn58	9.065	122.8
Cys58	8.181	122.2	Cys59	8.169	122.2
Pro59			Pro60		
Lys60	8.336	119.2	Lys61	8.356	119.1
Thr61	7.909	114.4	Thr62	7.905	114.7
Gln62	8.003	121.2	Gln63	8.025	121.6
Thr63	7.894	115.2	Thr64	7.889	115.1
Gln64	7.855	125.6	Gln65	7.851	125.5
Thr65			Thr66		
Ser66			Ser67		
Ala67			Ala68		
Lys68	8.190	121.1	Lys69		
Gly69	8.055	109.5	Gly70	8.044	109.3
Cys70	8.303	122.5	Cys71	8.277	123.5
Thr71	9.221		Thr72	9.248	124.3
Cys72			Cys73	9.848	125.7
Gly73	8.675	109.1	Gly74	8.666	109
Glu74	8.411	119.1	Glu75	8.406	119.1
Gly75	8.749	110.5	Gly76	8.748	110.5
Cys76	7.252	127.6	Cys77	7.181	122.8
Thr77			Thr78	8.255	119.9
Cys78	8.731	129.2	Cys79	8.716	129.2
Ala79	9.037	132.2	Ala80	9.002	131.7
Ser80	8.614	115.8	Thr81	8.396	117.4
Cys81	8.090		Cys82	8.501	124.6
Ala82	7.611	123.1	Ala83	7.709	121.5
Thr83	7.647	118.7	Ala84	7.328	127.9

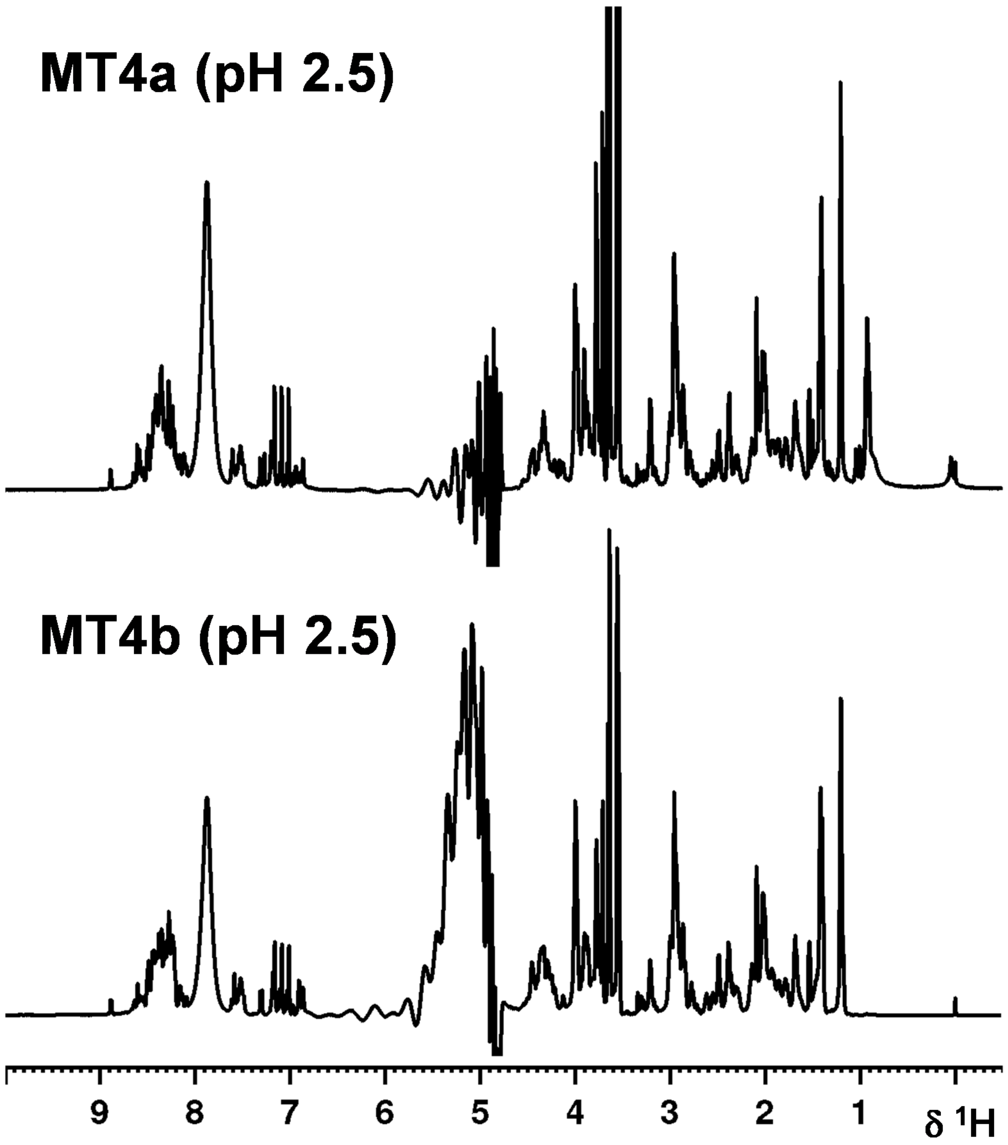

Figure S1. Full 1D ^{1}H spectra of MT4a and MT4b at pH 2.5, demonstrating the complete unfolding of the proteins as judged by the lack of chemical shift dispersion for backbone amide protons and absence of high-field-shifted methyl protons below 1 ppm.

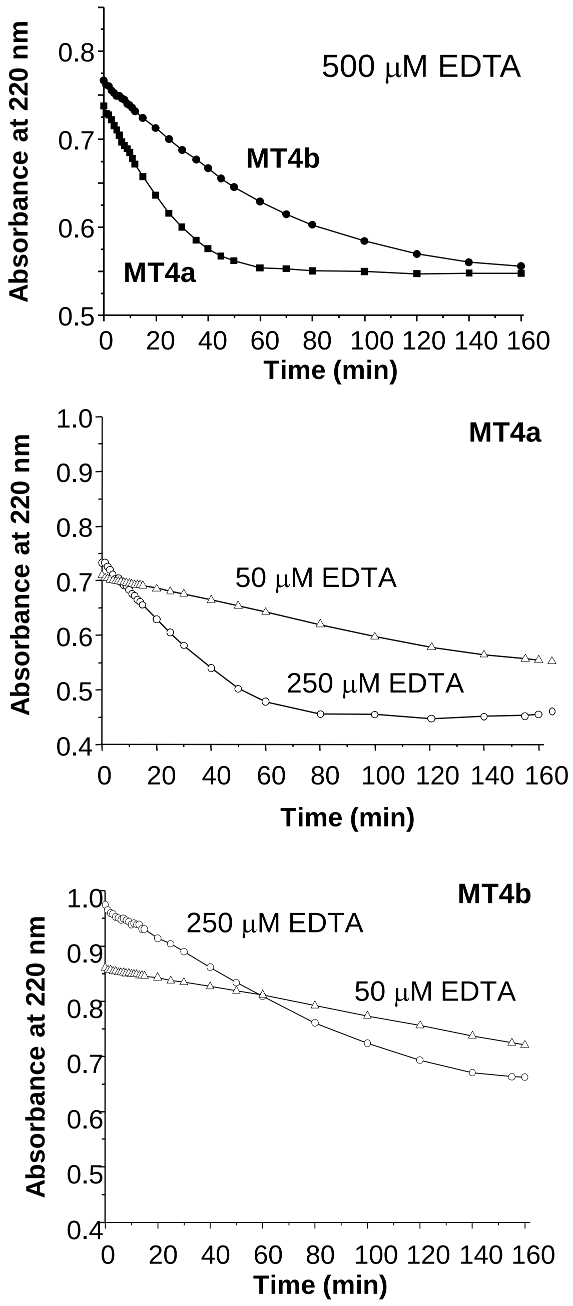

Figure S2. Timecourse data for the reaction of Zn_6MT4a and Zn_6MT4b with different excesses of EDTA (5 μM protein, 25 mM Tris-Cl buffer, pH 7.3).

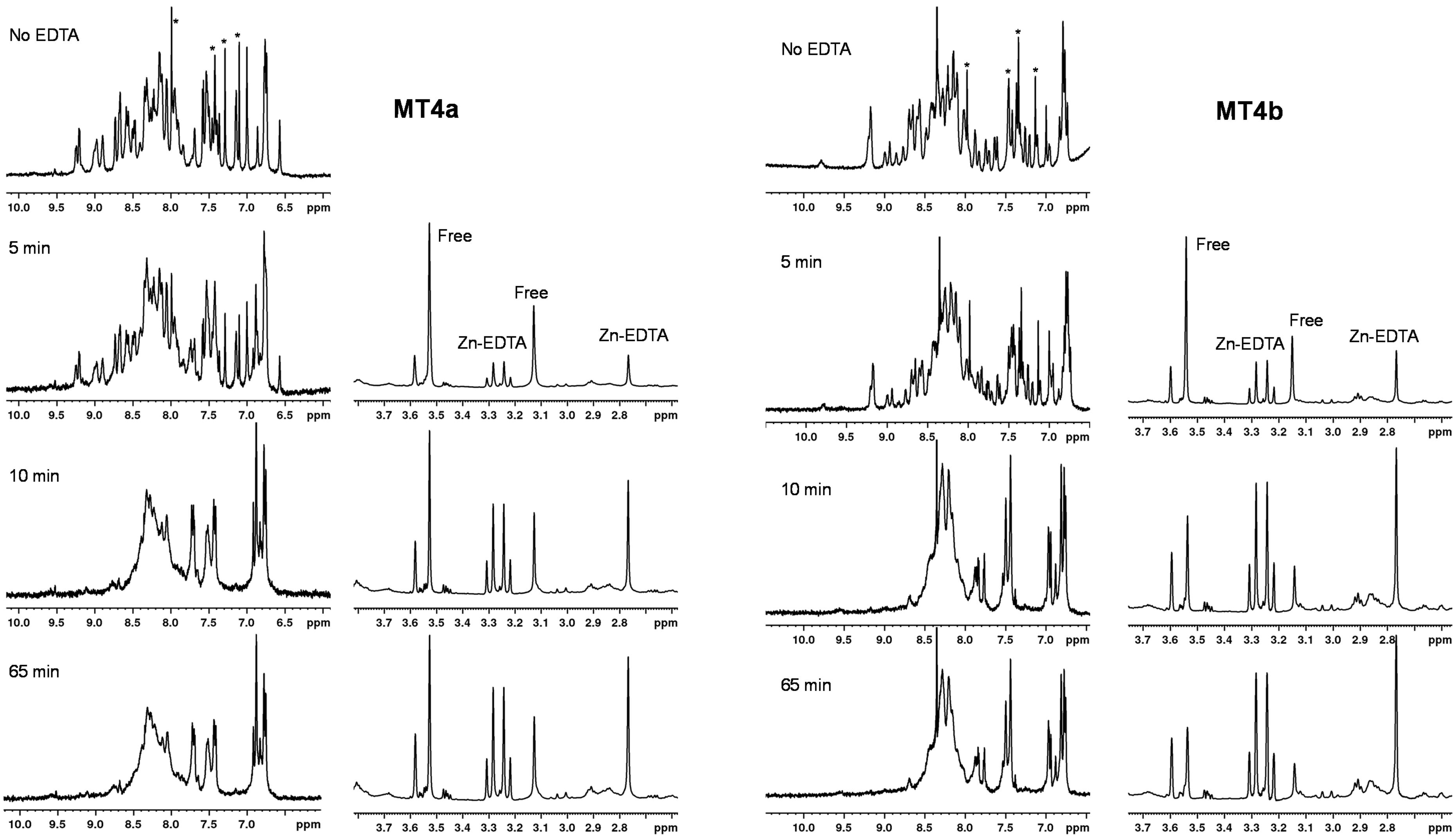

Figure S3. Selected regions of 1D ^{1}H NMR spectra during reaction of Zn_6MT4a and Zn_6MT4b with EDTA, showing increasing unfolding alongside increasing formation of the Zn-EDTA complex (50 mM Tris-D_{11}, 50 mM NaCl, 10 % D_2O, pH 7.4). The peaks marked with asterisks correspond to the Hε1 and Hδ2 protons of His42/43 and His50/51.

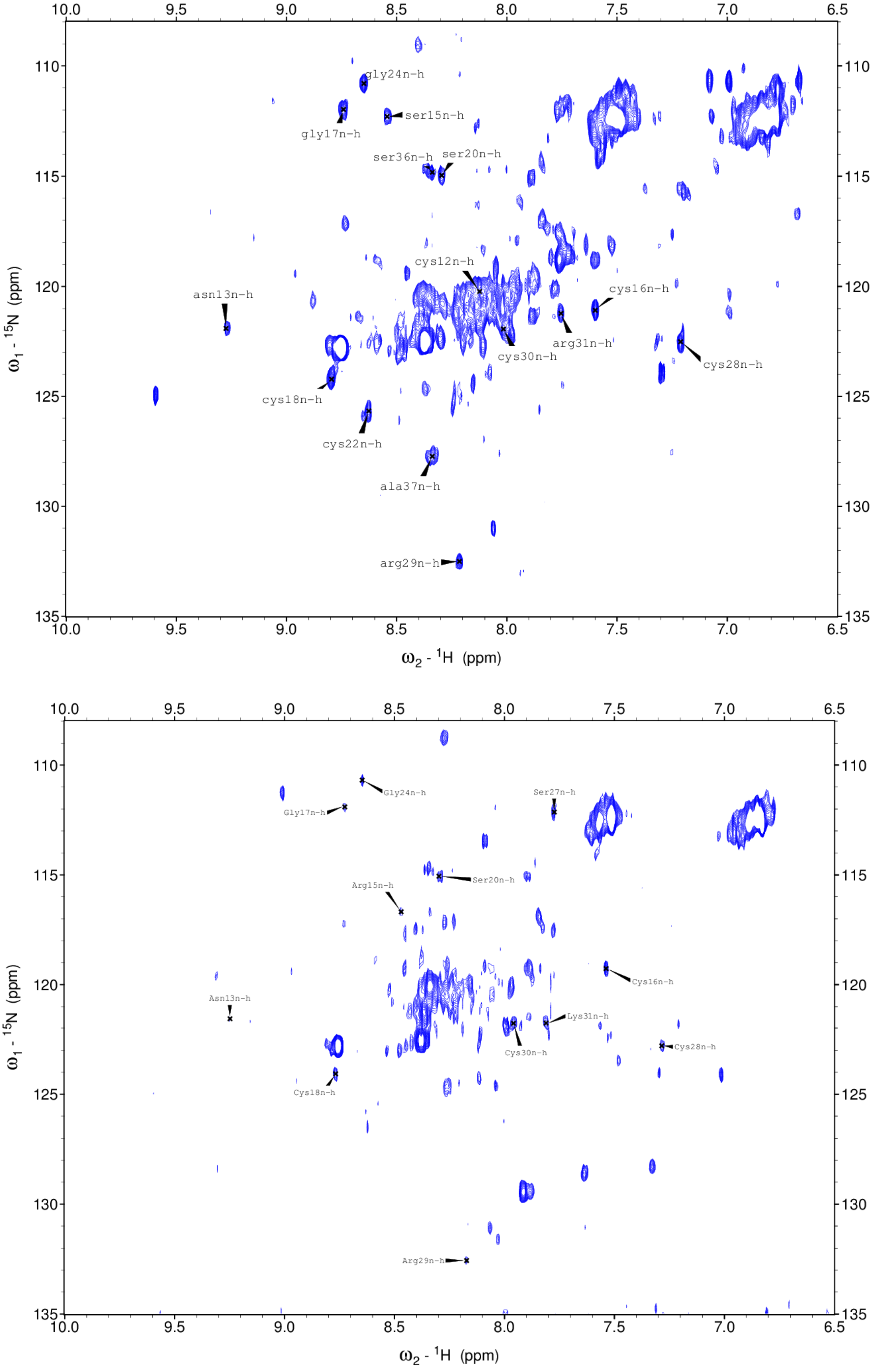

Figure S4. [^{1}H,^{15}N] HSQC spectrum of MT4a (top) and MT4b (bottom) after overnight (> 8 hours) reaction with an equimolar amount (with respect to [Zn]) of EDTA. Many resonances for domain I residues are clearly discernible. The very strong peaks present in both spectra in the central region are due to a contamination with a small molecule; those in the upper right corner are from side-chain amide NH_2 groups.
